# Disease prevalence and number of health care visits among members of a nationwide sports organization compared to matched controls

**DOI:** 10.1186/s12889-021-10466-9

**Published:** 2021-03-06

**Authors:** Hanna Lindblom, Mats Lowén, Tomas Faresjö, Kristofer Hedman, Per Sandström

**Affiliations:** 1 Department of Health, Medicine and Caring Sciences, Division of Prevention, Rehabilitation and Community Medicine, Unit of Physiotherapy, Linköping University, Linköping, Sweden; 2 Unit for Public Health and Statistics, Department of Health, Medicine and Caring Sciences, Linköping University, Linköping, Sweden; 3 Department of Health, Medicine and Caring Sciences, Division of Prevention, Rehabilitation and Community Medicine, Linköping University, Linköping, Sweden; 4 Department of Clinical Physiology, and Department of Health, Medicine and Caring Sciences, Linköping University, Linköping, Sweden; 5 Department of Surgery, Department of Biomedical and Clinical Sciences, Linköping University, Linköping, Sweden

**Keywords:** Physical inactivity, Lifestyle, Training effects

## Abstract

**Background:**

Physical activity has positive effects on several diseases and may reduce the risk of morbidity and the mortality rate. Whether the prevalence of disease and health care consumption differ between the members of sports organizations and the general population has not been established. Hence, this pilot study aimed to compare the prevalence of diseases known to be associated with physical inactivity and health care consumption in members of a large non-profit sports organization and an age-, sex- and geographically matched random sample from the general population.

**Methods:**

Subjects in two Swedish cities who exercised at least once a week and had been members for at least two years in the non-profit sports organization Friskis&Svettis were invited. A randomized age-, sex- and geographically matched sample was drawn from the general population. Data on disease prevalence (by International Statistical Classification of Diseases and Related Health Problems, Tenth Revision (ICD-10) codes) and health care consumption were retrieved using the members’ personal identification numbers through a regional health care database. Between-group differences in the prevalence of disease were compared using chi^2^-tests and logistic regression between members and controls. Health care consumption was defined as the number of visits, stratified by primary and hospital care, and was compared using chi^2^-tests and Mann-Whitney U-tests.

**Results:**

In total, 3015 subjects were included in each group (response rate 11%). Controls had higher prevalence rates of musculoskeletal diseases (13.3% vs. 11.6%, *p* = 0.047), metabolic disease (10.4% vs. 5.4%, *p* < 0.001), hypertension (16.6% vs. 11.7%, *p* < 0.001), psychiatric diseases (8.9% vs. 7.1%, *p* = 0.012) and lung cancer (0.4% vs. 0%, *p* = 0.001) than the members. The total number of health care contacts was 22% higher in the controls than in the members, whereas the proportion of subjects with at least one health care visit was larger in the members (89% vs. 79%, *p* < 0.001).

**Conclusions:**

The prevalence rates of lifestyle diseases related to musculoskeletal, metabolic and psychiatric diseases, hypertension and lung cancer, and the overall health care consumption, were lower among members of a sports organization than among controls. However, longitudinal studies are needed to establish a cause-effect relationship between membership and disease development.

**Supplementary Information:**

The online version contains supplementary material available at 10.1186/s12889-021-10466-9.

## Background

Physical activity is well known for its positive effects on psychiatric, neurological, metabolic, cardiovascular, pulmonary, and musculoskeletal diseases and cancer [1]. Higher levels of physical activity, irrespective of intensity, and less sedentary time are associated with lower rates of premature all-cause mortality in middle-aged or older adults [2]. There is a dose-response relationship with maximal risk reductions in people engaging in up to 375 min/day of light-intensity physical activities or 24 min/day of moderate- to vigorous-intensity physical activities across the US and western European countries [2]. Physical activity of moderate to vigorous intensity has also been shown to reduce the risk of morbidities requiring hospital care, particularly for cardiovascular diseases [3]. Among the physically inactive, health care utilization is more common than among the physically active [4]. Even though a trend of decreasing aerobic capacity has been observed over the last 20 years in Sweden [5], engagement in organized leisure-time physical activity and membership in non-profit and commercial gyms have increased [6, 7]. Whether the prevalence of disease or the health care consumption is different between members of these gyms and the general population has not been studied thus far.

The aim of this pilot study was to compare the prevalence rates of diseases known to be associated with physical inactivity and health care consumption in members of a large, nationwide non-profit sports organization and an age-, sex- and geographically matched random sample from the general population.

## Methods

In this cross-sectional pilot study, we utilized gym membership registry data to administer questionnaires and to crosslink data with health care registry data using the unique personal identification numbers connected to every Swedish citizen. In Sweden, the health care system is publicly financed through taxes including a general health care insurance for all citizens with health care visits with low fees or free of charge for all necessary care for all Swedes. Contacts with the public health care are usually initiated via the primary health care, that may refer patients to hospital and specialized care when necessary [8]. Visits to legitimized health care professionals result in documentation in the patients’ medical records. A regional health care register, that was used in the present study, covers details on all health care utilization including diagnoses registered in the medical records, but no demographic or socioeconomic data about the patients.

### Non-profit sports organization

In Sweden, a northern European country with ~ 10.3 million inhabitants, 575,430 (6%) are members of a non-profit organization with national reach called Friskis&Svettis [9]. The purpose of Friskis&Svettis, founded in 1978, is to provide accessible and fun means to be physically active. Originally based on indoor and outdoor aerobic-style classes, today, Friskis&Svettis provides members with a wide range of physical activities, including individual gym training and different classes. Most classes aim at aerobic exercise at a moderate to vigorous intensity. Friskis&Svettis is organized into 109 individual sports clubs spread throughout Sweden, 42 sports clubs in Norway and a few in European cities, including Brussels, Paris and London [9]. The sports clubs are present in both cities and at the countryside and own their own facilities, rent facilities specifically planned and equipped for their needs (most often) or rent facilities that are used by schools at daytime, with the aim of being close to their members. In the current study, two Swedish individual Friskis&Svettis sports clubs from the two neighbouring and equally sized Swedish cities Linköping and Norrköping (population ~ 150,000 in each county) were included. In each county, ~ 10% of the inhabitants were members of the respective Friskis&Svettis sports club. Costs differ between different sports clubs but are generally low. In the areas in the present study a one-year membership cost 261–345€, representing 6–8‰ of the mean annual income in Sweden in 2019.

### Participants

**Fig. 1:**
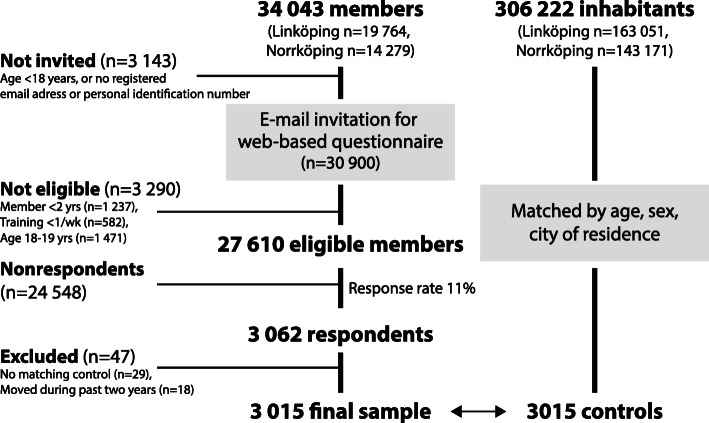
Study flowchart

Members age 20 or older in either of the two neighbouring Friskis&Svettis sports clubs were invited to take part in the study in November 2019 (Fig. 1, Study flow chart). Members who registered without their personal identification number or e-mail address were not invited to take part in the study. Subjects who had been a member for at least two years and reported training at least once a week on average were considered eligible (*n* = 27,610).

To account for known sociodemographic differences between the two cities [10], control subjects were randomly sampled from the general populations in Linköping and Norrköping separately, using 1:1 age (birth year and month) and sex matching. No data on physical activity level, other lifestyle habits, or on membership in any sports organization were available for control subjects. Controls were not actively involved in the study and were not informed about their participation.

### Data collection

#### Questionnaire

All members of Friskis&Svettis in the two cities received an e-mail in November 2019 inviting them to take part in the study. Three reminders were sent in the week following the original invitation. The e-mails linked to a webpage providing detailed information about the study and how personal data were handled. If a subject consented, he or she was asked a) how long they had been a member of Friskis&Svettis (in total) and b) how many times per week they exercised (at Friskis&Svettis or elsewhere). Pre-specified alternatives for each question were used (Additional file 1).

#### Diagnoses and health care consumption

The personal identification number for each eligible, consenting member was retrieved from the Friskis&Svettis membership database and used to extract data on disease prevalence and health care consumption (i.e., number of health care visits) using regional health care data, in turn incorporating medical record data from all public and a majority of private primary care units and from all hospital units. The same data were retrieved for the random population sample of control subjects.

As per the International Statistical Classification of Diseases and Related Health Problems, Tenth Revision (ICD-10) codes, the prevalence of a priori selected specified diseases (Additional file 2) was determined. An ICD-10 code in the registry coded for any health care professional-administered care between January 1st 2018, and December 31st 2019 was used to define disease prevalence. Diagnostic codes from primary care, outpatient hospital care (to medical specialists) or inpatient hospital care were considered. In addition, the profession of the respective health care provider at each recorded visit was retrieved. Any health care visit that was registered in the patients’ medical records with the specified diagnostic codes was considered as prevalence of disease regardless of whether the disease was cured or not. Diagnoses recorded by private care providers without a care agreement with the public health care system were not considered. However, in Sweden, the vast majority of health care contacts are made within the public health care system, or within clinics affiliated with the public health care system, for the diagnoses specified in this study.

In brief, we considered the following diseases with a known or suspected association with physical inactivity: neoplasms (cancers of the lung, breast, gastrointestinal tract, urogenital system), metabolic disease (type 2 diabetes mellitus, obesity, dyslipidaemia), hypertension, coronary artery disease, psychological disease (anxiety, depression), dementia, and musculoskeletal disease (osteoporosis, osteoarthritis, fractures [hip or shoulder], back pain).

### Statistical analysis

No a priori sample size calculation was made in this pilot study, as preliminary data were lacking. The prevalence of disease was determined as the ratio between the number of cases with the specific diagnostic code(s) and the number of all potential cases in the respective cohort. The chi^2^-test was used to analyse differences in prevalence of diagnostic codes in members vs controls. Logistic regression was used to calculate the odds ratio (OR) with a 95% confidence interval of having a disease for respondents who were members compared to respondents in the control group.

The number of health care visits was compared between groups using the Mann-Whitney U-test, and the proportion of subjects with at least one visit was compared using chi^2^-tests. When comparing the number of health care visits among members with different training frequencies, one-way ANOVAs and the Kruskal-Wallis test were used.

A two-sided *p*-value < 0.05 and a 95% CI not including 1 were considered statistically significant. Analyses were performed with R Studio v1.1.456 (R Studio Inc., Vienna, Austria) and SPSS v25.0 (IBM Corp, Armonk, NY, USA). We did not adjust for numeral testing since this was a hypothesis generating study, where we aimed to explore all possible associations within the data.

## Results

### Subjects

**Fig. 2:**
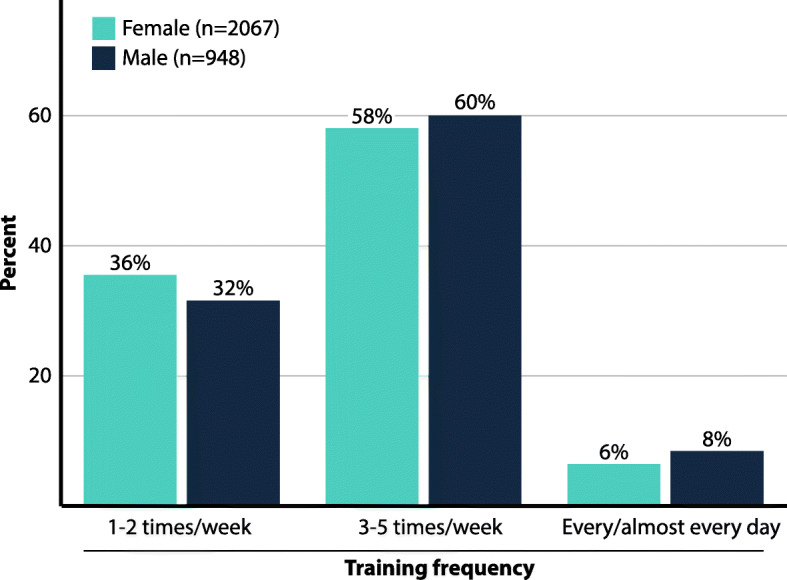
Exercise pattern among Friskis&Svettis members

In total, *n* = 3062 of the *n* = 27,610 eligible members of Friskis&Svettis responded to the web questionnaire (response rate 11%, Fig. 1). Members who had moved or for whom no matching control could be identified were excluded from analysis. In total, *n* = 3015 subjects (*n* = 2067 [69%] female) were included in each group, matched for age (mean age 53 ± 15 years, range 20–92 years). Among the members, both male and female, the most frequent training pattern was exercising 3–5 times/week, followed by 1–2 times per week (Fig. 2). Overall, 40% of the members had been members more than 10 years, while 23% had been members only during the last 2–3 years.

### Prevalence of disease

Subjects in the control group had higher prevalence rates of musculoskeletal disease (13.3% vs. 11.6%, *p* = 0.047), metabolic disease (10.4% vs. 5.4%, *p* < 0.001), hypertension (16.6% vs. 11.7%, p < 0.001), psychiatric disorders (8.9% vs. 7.1%, *p* = 0.012) and lung cancer (0.4% vs. 0%, *p* = 0.001) than members (Table 1). Disease prevalence rates per age, sex and group are presented in Additional files 3 and 4.

**Table 1:** Disease prevalence rates and odds ratios for members having the respective disease compared to controls

	Controls^**a**^ (***n*** = 3015)	Members^**a**^ (n = 3015)	P (Chi^**2**^)	Odds ratio(95% CI)
Musculoskeletal disease	401 (13.3%)	350 (11.6%)	0.047	0.86 (0.74–1.00)
Metabolic disease	314 (10.4%)	163 (5.4%)	< 0.001	0.49 (0.40–0.60)
Hypertension	500 (16.6%)	354 (11.7%)	< 0.001	0.67 (0.58–0.78)
Coronary artery disease	20 (0.7%)	17 (0.6%)	0.62	0.85 (0.44–1.62)
Psychiatric disease	267 (8.9%)	214 (7.1%)	0.012	0.79 (0.65–0.95)
Dementia	8 (0.3%)	5 (0.2%)	0.41	0.62 (0.20–1.91)
Lung cancer	11 (0.4%)	0 (0.0%)	0.001	N/A
Breast cancer	14 (0.5%)	14 (0.5%)	1.0	1.00 (0.48–2.10)
GI-cancer	9 (0.3%)	3 (0.1%)	0.08	0.33 (0.09–1.23)
Urogenital cancer	10 (0.3%)	4 (0.1%)	0.11	0.40 (0.13–1.27)

a, Mean age of both groups: 53 ± 15 years. Abbreviations: GI gastrointestinal.

GI-cancer was defined as cancer of the colon, rectum, oesophagus, stomach or liver; Urogenital cancer was defined as cancer of the kidney or urinary bladder; Metabolic disease was defined as a diagnosis of obesity, dyslipidaemia or type 2 diabetes mellitus; Psychiatric disease was defined as a diagnosis of depression or anxiety disorder; Musculoskeletal disease was defined as back pain; osteoporosis with or without fracture; a fracture of the femur, upper arm or shoulder; or arthrosis of the knee or hip

### Health care consumption

**Fig. 3:**
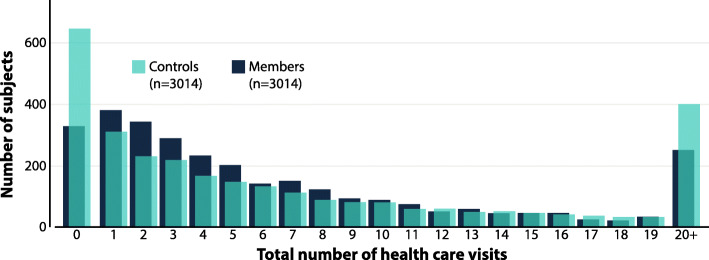
Total number of health care contacts in two years in controls (turquoise) and members (dark)

The total number of health care visits was 22% higher among controls than among members (27,658 vs. 22,735). In contrast, the proportion of subjects with at least one health care visit was larger among members than among controls (89% vs. 79%, *p* < 0.001). The distribution of the total number of visits is displayed in Fig. 3. Comparisons between those with no visit and those with 1–5 visits are displayed in additional file 5. As per type of health care visit (Table 2), a larger proportion of members than controls had 1–9 visits to primary care (68% vs. 58%, p < 0.001) and to outpatient hospital care (62% vs. 50%, p < 0.001). For inpatient care, control subjects had more than twice the number of visits (*n* = 671 vs. *n* = 295), and the proportion of control subjects having at least 2 recorded inpatient care visits was considerably larger than in members (4% vs. 1.5% of subjects, p < 0.001). When comparing the subjects with 1–20 visits with those with 20+ visits, among the 4460 subjects with one to 20 health care contacts in total, 44% of contacts were to primary care, while 54 and 2% of contacts were to outpatient and inpatient hospital care (Additional file 6). In the 597 subjects with more than 20 health care contacts (range 21–243), 26% were to primary care while 71 and 2% were to outpatient and inpatient hospital care. In total, 12 subjects (0.002%) had more than 100 health care contacts in total (excluding the outlier with > 500 contacts, not included in the analysis). Of these 12 (median age 42 years, three male), seven were controls and five were members.

**Table 2:** Health care visits per group and type of health care setting

	Controls (***n*** = 3014)	Members (*n* = 3014)	*P*-value
**Primary care visits**			
Sum of visits, n	10,058	8210	–
Mean ± SD	3.34 ± 6.41	2.72 ± 4.04	0.67^a^
Subjects with 1–9 visits, n (%)	1743 (58%)	2047 (68%)	< 0.001^b^
Subjects with ≥10 visits, n (%)	252 (8%)	144 (5%)	< 0.001^b^
**Outpatient hospital care visits**			
Sum of visits, n	16,929	14,230	–
Mean ± SD	5.62 ± 11.61	4.72 ± 8.79	0.002^a^
Subjects with 1–9 visits, n (%)	1509 (50%)	1870 (62%)	< 0.001^b^
Subjects with ≥10 visits, n (%)	492 (16%)	382 (13%)	< 0.001^b^
**Inpatient hospital care visits**			
Sum of visits, n	671	295	–
Mean ± SD	0.22 ± 0.81	0.10 ± 0.41	< 0.001^a^
Subjects with 1 visit, n (%)	270 (9%)	177 (6%)	< 0.001^b^
Subjects with ≥2 visits, n (%)	122 (4%)	46 (1.5%)	< 0.001^b^

a, Mann-Whitney U-test; b, Chi^2^-test. One outlier control subject with > 500 health care visits over two years and the matching member were removed from the total sample of 3015 subjects per group. Primary care visits correspond to visits to a non-hospital based health central (typically to a general practitioner), while outpatient hospital visits typically represent visits to medical specialists at hospital clinics

The numbers of visits per type of health care professional among controls and members are presented in Additional file 7. In a subgroup analysis per training frequency, the total number of health care contacts was lower among members in all groups than among control subjects (Table 3**)**. However, there were no significant differences in the number of health care visits depending on training frequency.

**Table 3:** Health care visits per exercise training frequency vs controls

	Exercise training group per training frequency				Controls (*n* = 3014)
	1–2/week (***n*** = 1032)	3–5/week (***n*** = 1769)	Almost every day (***n*** = 213)	*P*-value	
Male: Female (n)	298:734	569:1200	80:133	0.03^a^	948:2066
Age (mean ± SD)	55.3 ± 14.8	53.1 ± 15.5	47.5 ± 16.5	< 0.001^b^	53.4 ± 15
**Primary care visits**					
Mean (SD)	2.93 ± 4.71	2.64 ± 3.70	2.39 ± 3.20	0.07^b^	3.3 ± 6.4
Subjects with 1–9 visits, n (%)	718 (70%)	1182 (67%)	147 (69%)	0.30^a^	1743 (58%)
Subjects with ≥10 visits, n (%)	53 (5%)	84 (5%)	7 (3%)	0.51	252 (8%)
**Hospital outpatient care visits**					
Mean (SD)	4.47 ± 7.27	4.81 ± 9.65	5.23 ± 7.99	0.19^b^	5.6 ± 11.6
Subjects with 1–9 visits, n (%)	639 (62%)	1096 (62%)	135 (63%)	0.92^a^	1509 (50%)
Subjects with ≥10 visits, n (%)	134 (13%)	214 (12%)	34 (16%)	0.26	492 (16%)
**Hospital inpatient care visits**					
Mean (SD)	0.09 ± 0.35	0.10 ± 0.43	0.11 ± 0.52	0.64^b^	0.22 ± 0.81
Subjects with 1 visit, n (%)	65 (6%)	107 (6%)	5 (2%)	0.07^a^	270 (9%)
Subjects with ≥2 visits, n (%)	12 (1%)	27 (1.5%)	7 (3%)	0.07	122 (4%)

a, Chi^2^; b, One-way ANOVA. P-value denotes difference between training frequency groups. One outlier control subject with > 500 health care visits over two years and the matching member were removed from the total sample of 3015 subjects per group. Primary care visits correspond to visits to a non-hospital based health central (typically to a general practitioner), while outpatient hospital visits typically represent visits to medical specialists at hospital clinics

## Discussion

The main results of this pilot study were as follows: (1) active members in a non-profit sports organization had lower frequencies of musculoskeletal and metabolic diseases, hypertension, psychiatric diseases and lung cancer than an age-, sex- and geographically matched control group; (2) active members also had lower health care consumption in total, and especially considering inpatient hospital care; and (3) a larger proportion of active members had 1–9 visits to primary care or hospital outpatient care than the general population.

### Physical activity as prevention and treatment of disease

The beneficial effects of physical activity for the treatment and prevention of a multitude of diseases are well established [1, 2]. In addition, physical activity of moderate to vigorous intensity reduces the risk of morbidities requiring hospital care [3]. For example, physical activity is considered a first-line treatment for osteoarthritis [11, 12] and seems promising as a primary or adjunctive therapy in the prevention and treatment of osteoporosis as well as in prevention of falls [13]. In light of this, our finding of a lower prevalence of musculoskeletal disease among members than in controls could imply an under-utilization of physical activity as a preventive measure in these subjects. Positive effects of physical activity have already been observed with small doses of physical activity [14], and it is possible that the members had all experienced these effects since only members active at least once per week were included. This may also explain why no differences in the number of health care visits were seen between members exercising the least and the most often.

We found lower prevalence rates of metabolic diseases and hypertension among the members than among the controls from the general population. This could either reflect the beneficial effect of regular physical activity on these diseases [15, 16], associated with being an active member of a sports organization, or indicate that patients with these diseases do not become members of similar organizations as frequently. Additionally, one must consider that positive lifestyle habits often appear in combination [17, 18], with the possibility that more members engaged in physical activity, maintained a healthy diet and did not smoke. Previous reports have pointed out that in many cases, care for metabolic disease is still predominantly focused on medical treatment [19]. Since these are complex diseases affected by lifestyle habits, motivation and support for behavioural change may also be paramount [20] for the success of long-term lifestyle changes.

Reduced physical activity may be a long-term consequence of depression [21], which, together with the positive effects of physical activity on symptoms of depression and anxiety, may explain the differences in the prevalence of mental disorders between members and controls. In a longitudinal Swedish study in women, higher depression scores also predicted lower levels of physical activity over time [21]. In anxiety, this behaviour often co-exists with depression, and physical activity may be protective against the development of symptoms and may reduce present symptoms [22]. We found a lower prevalence of lung cancer (0 vs. 11 cases) in members than in controls, while the prevalence of other cancer types was similar between groups. Although longitudinal data provide evidence for relative risk reductions of 10–20% in highly active people compared to those less active for bladder, breast, colon, endometrial, oesophagus, renal and gastric cancers [23], this was not supported in the present study, which could reflect a lack of statistical power for these relatively rare diseases or the fact that we retrieved medical record data only for the past two years. Subjects with recently diagnosed cancer may experience symptoms and treatment effects preventing active participation in leisure time and organized physical activities. As a dose-response relationship has been shown between higher physical activity levels and a lower risk of several cancers [23], a larger sample size permitting stratification into exercise frequency would be of value.

Overall, this study highlights a need for sports organizations and the health care system to further develop strategies to reach patient groups with either an increased risk for developing or already manifesting disease, in combination with information to the general population about the beneficial effects of physical activity. While the evidence for physical activity as medicine is clear [2], there is a need for longitudinal, large-scale studies on the effects of interventions to increase the level of physical activity, especially in patient groups at risk. Joint efforts between non-profit or commercial sports organizations/gyms, the healthcare system and researchers may be one possible way forward, as proposed in the current pilot study.

### Health care consumption

The total health care consumption, measured as the number of health care visits, was lower among members than among controls. However, the proportion of individuals not seeking primary or outpatient hospital care at all was higher among controls. Earlier studies in a Swedish context have shown that health care consumption differs depending on socioeconomic group and health status, with lower income groups abstaining from seeking care to a greater extent [24]. Generally, people with higher socioeconomic status are more physically active in their leisure time [25, 26] but are less active with housework and care [25]. Hence, it is possible that our results reflect a difference in health care consumption due to a different health-seeking behaviour rather than a difference in disease prevalence. This is supported by the lower frequency of prevalent disease among the members, despite a larger proportion visiting health care providers. The fact that the controls had more inpatient hospital care visits may further support this hypothesis. Hence, the greater proportion of members than controls with 1–9 visits to primary care and outpatient care, may indicate that the members are more health conscious and proactive, doing regular check-ups to prevent serious health issues. If disease develops, its detection and treatment may also commence earlier and with greater success rates in people who do regular check-ups, such as the members. Early detection, at low cost, may also reduce the need for inpatient care or lengthy treatments, which the results of the present study may also indicate.

Over the years, hospitalizations due to physical inactivity have decreased in Sweden, whereas the amounts of outpatient hospital care and primary care visits have increased [27]. Physical inactivity causes both morbidity and mortality and is a major economic burden across the world [28]. The health care costs due to physical inactivity in Sweden in 2016 were 1.7 billion SEK [27], which places further emphasis on the importance of physical activity for the prevention and treatment of diseases, where sports organizations may play important roles. Organizations with low costs, such as Friskis&Svettis, that also have the potential to reach lower socioeconomic groups may be particularly beneficial since access to and resources for training have been reported as major barriers for physical activity in these groups [25].

### Limitations

First, the cross-sectional design precludes any conclusions on causality; thus, we are unable to determine whether being an active member reduces risk of disease or whether subjects with developed disease are unable to participate in training. Additionally, one must consider that the treatment of diseases, such as lung cancer, may prevent or complicate training during the active phase of treatment. Second, there may also be an underestimation of less severe conditions that do not require yearly health care visits since these might not have rendered a health care visit within the two-year time frame of this study. Third, the response rate (11%) was low. However, when comparing across the ~ 30,000 overall possible respondents, the age and sex distributions were representative. Fourth, as we lack data on physical activity for the control group, this group most likely includes active individuals, and we cannot draw conclusions on the beneficial effects of physical activity per se. Finally, we lack data on lifestyle habits and socioeconomic status for both groups. Hence, future studies using national registries with more data on each individual are important to respond to specific questions related to the influence of lifestyle, physical characteristics and socioeconomic status on health and diseases among physically active versus controls.

### Strengths

This pilot study was unique in using a nationwide, non-profit sports organization that reaches a large proportion of Swedes. The design was feasible and could easily be scaled up to reach 10- to 20-fold as many subjects. Being a non-profit organization, membership is available at a fairly low cost, and many Swedish employers contribute financially to wellness activities among their employees, making it possible to reach both low- and high-income groups. The results of this study are supported by using controls taken from the general population and a fairly large sample. By including subjects from two neighbouring cities with marked differences in cardiovascular disease occurrence, longevity and socioeconomic profile, we further increased the generalizability of the findings [10, 29]. Finally, using participants’ Swedish personal identification numbers and cross-linking with regional health care data allowed almost full coverage of diagnoses during the two years investigated.

## Conclusions

In the current pilot study, active participants in a non-profit sports organization had lower prevalence of lifestyle-related diseases and an overall lower health care consumption than a random, matched sample of the general population. Larger studies are needed to establish whether the same is true for rare diseases, taking into account socioeconomic factors, lifestyle habits and more detailed data on exercise patters. Additionally, a research design rendering longitudinal data is required for establishing a cause-effect relationship between training habits and health care consumption.

## Supplementary Information

**Additional file 1.** The questionnaire for the members

**Additional file 2.** Diagnoses included in the disease prevalence comparisons between members and controls

**Additional file 3.** Prevalence of disease per age group and sex in males.

**Additional file 4.** Prevalence of disease per age group and sex in females.

**Additional file 5.** Comparison between subjects with no or with one to five total number of health care visits over the two-year study period.

**Additional file 6.** Comparison between subjects with 1–20 or more than 20 total number of health care visits over the two-year study period.

**Additional file 7.** Health care contacts per group and the type of health care provider profession.

## Data Availability

All data generated or analyzed during this study are included in this published article.
