# Supplementary material for: Disease prevalence and number of health care visits among members of a nationwide sports organization compared to matched controls
Source: BMC Public Health. 2021 Mar 6;21:455. doi: 10.1186/s12889-021-10466-9 (PMC7937278; doi:10.1186/s12889-021-10466-9)
Supplement: Supplementary file 1 — Additional file 1. The questionnaire for the members [file 12889_2021_10466_MOESM1_ESM.docx]

| **Additional file 1. The questionnaire for the members** | | | | | |
| --- | --- | --- | --- | --- | --- |
| **Questions** | **Responses** | | | | |
| When looking back, how many years have you been a member of Friskis&Svettis Linköping/Norrköping in total? | Less than 2 years | 2-3 years | 4-6 years | 7-10 years | More than 10 years |
| How often do you do any exercise (at Friskis&Svettis or elsewhere)? | Every, or almost every day | 3-5 times/week | 1-2 times/week | A couple of times/month | More seldom |
| The questionnaire was delivered to participants only in Swedish. The authors’ translation is provided above. | | | | | |
