# Supplementary material for: Disease prevalence and number of health care visits among members of a nationwide sports organization compared to matched controls
Source: BMC Public Health. 2021 Mar 6;21:455. doi: 10.1186/s12889-021-10466-9 (PMC7937278; doi:10.1186/s12889-021-10466-9)
Supplement: Supplementary file 2 — Additional file 2. Diagnoses included in the disease prevalence comparisons between members and controls [file 12889_2021_10466_MOESM2_ESM.docx]

| **Additional file 2. Diagnoses included in the disease prevalence comparisons between members and controls** | |
| --- | --- |
| **ICD-10* chapter** | **ICD-10 codes** |
| Neoplasms (Cancers) | **Gastrointestinal**   - Oesophagus (C15.X) - Stomach (C16.0X) - Colon (C18.X, C19.X) - Rectum (C20.X) - Liver (C22.X)   **Lung** (C34.9A-G) **Breast, post-menopausal** (C50.X)  **Urogenital**   - Kidney (C64.X) - Bladder (C67.X) |
| Endocrine, nutritional and metabolic diseases | **Metabolic**   - Diabetes mellitus, type 2 (E11.X) - Obesity (E66.X) - Dyslipidaemia (E78.0-E78.5) |
| Mental, behavioural and neurodevelopmental disorders | **Psychological**   - Anxiety (F41.X) - Depression (F32.X, F33.X)   **Dementia** (F00.X, F01.X, F03.X) |
| Diseases of the circulatory system | **Hypertension** (I10.X)  **Coronary artery disease** (I20.X) |
| Diseases of the musculoskeletal system and connective tissue  Injury, poisoning and certain other consequences of external causes | **Musculoskeletal**   - Osteoporosis (M81.X, M80-0X) - Osteoarthritis (M17.X, M16.X) - Back pain (M54.X) - Fractures (S72.X, S42.X, S52) |
| *ICD-10, International Statistical Classification of Diseases and Related Health Problems, Tenth Revision. The analyses in the present study were primarily made for the diagnoses and diagnostic groups in bold. | |
