# Supplementary material for: Disease prevalence and number of health care visits among members of a nationwide sports organization compared to matched controls
Source: BMC Public Health. 2021 Mar 6;21:455. doi: 10.1186/s12889-021-10466-9 (PMC7937278; doi:10.1186/s12889-021-10466-9)
Supplement: Supplementary file 3 — Additional file 3. Prevalence of disease per age group and sex in males. [file 12889_2021_10466_MOESM3_ESM.docx]

| **Additional file 3. Prevalence of disease per age group and sex in males.** | | | | | | | | | | |
| --- | --- | --- | --- | --- | --- | --- | --- | --- | --- | --- |
|  | **18-35** | | **35-50** | | **51-60** | | **61-70** | | **>70** | |
|  | **Controls** **(n=137)** | **Members** **(n=137)** | **Controls** **(n=184)** | **Members** **(n=184)** | **Controls** **(n=214)** | **Members** **(n=214)** | **Controls** **(n=235)** | **Members** **(n=235)** | **Controls**  **(n=178)** | **Members** **(n=178)** |
| **Musculoskeletal** | 3 (2%) | 7 (5%) | 13 (7%) | 11 (6%) | 30 (14%) | 19 (9%) | 35 (15%) | 28 (11%) | 16 (9%) | 30 (17%) |
| **Metabolic** | 0 | 2 (2%) | 6 (3%) | 3 (2%) | 22 (10%) | 9 (4%) | 55 (23%) | 26 (11%) | 36 (20%) | 38 (21%) |
| **Hypertension** | 0 | 0 | 10 (5%) | 7 (4%) | 34 (16%) | 22 (10%) | 76 (32%) | 53 (23%) | 66 (37%) | 73 (41%) |
| **Coronary** | 0 | 0 | 0 | 1 (0.5%) | 1 (0.5%) | 0 | 5 (2%) | 3 (1%) | 9 (5%) | 4 (2%) |
| **Psychiatric** | 9 (7%) | 13 (10%) | 8 (4%) | 16 (9%) | 10 (5%) | 11 (5%) | 11 (5%) | 13 (6%) | 8 (5%) | 5 (3%) |
| **Dementia** | 0 | 0 | 0 | 0 | 0 | 0 | 0 | 1 (0.4%) | 2 (1%) | 2 (1%) |
| **Lung cancer** | 0 | 0 | 0 | 0 | 0 | 0 | 3 (1%) | 0 | 2 (1%) | 0 |
| **Breast cancer** | 0 | 0 | 0 | 0 | 0 | 0 | 0 | 0 | 0 | 0 |
| **GI cancer** | 0 | 0 | 0 | 0 | 0 | 0 | 2 (1%) | 1 (0.4%) | 5 (3%) | 0 |
| **Urogenital cancer** | 0 | 0 | 0 | 0 | 1 (0.5%) | 1 (0.5%) | 2 (1%) | 1 (0.4%) | 2 (1%) | 0 |
