# Supplementary material for: Disease prevalence and number of health care visits among members of a nationwide sports organization compared to matched controls
Source: BMC Public Health. 2021 Mar 6;21:455. doi: 10.1186/s12889-021-10466-9 (PMC7937278; doi:10.1186/s12889-021-10466-9)
Supplement: Supplementary file 4 — Additional file 4. Prevalence of disease per age group and sex in females. [file 12889_2021_10466_MOESM4_ESM.docx]

| **Additional file 4. Prevalence of disease per age group and sex in females.** | | | | | | | | | | |
| --- | --- | --- | --- | --- | --- | --- | --- | --- | --- | --- |
|  | **18-35** | | **35-50** | | **51-60** | | **61-70** | | **>70** | |
|  | **Controls** **(n=370)** | **Members** **(n=370)** | **Controls** **(n=485)** | **Members** **(n=485)** | **Controls** **(n=538)** | **Members** **(n=538)** | **Controls** **(n=429)** | **Members** **(n=429)** | **Controls**  **(n=245)** | **Members** **(n=245)** |
| **Musculoskeletal** | 16 (4%) | 10 (3%) | 58 (12%) | 41 (9%) | 85 (16%) | 78 (15%) | 82 (19%) | 67 (16%) | 63 (26%) | 59 (24%) |
| **Metabolic** | 8 (2%) | 6 (2%) | 22 (5%) | 6 (1%) | 42 (8%) | 12 (2%) | 71 (17%) | 28 (7%) | 52 (21%) | 33 (14%) |
| **Hypertension** | 1 (0.3%) | 1 (0.3%) | 13 (3%) | 13 (3%) | 60 (11%) | 34 (6%) | 131 (31%) | 67 (16%) | 108 (44%) | 84 (34%) |
| **Coronary** | 0 | 0 | 0 | 0 | 0 | 2 (0.4%) | 2 (0.5%) | 4 (1%) | 3 (1%) | 3 (1%) |
| **Psychiatric** | 48 (13%) | 48 (13%) | 70 (14%) | 44 (9%) | 49 (9%) | 32 (6%) | 42 (10%) | 20 (5%) | 12 (5%) | 12 (5%) |
| **Dementia** | 0 | 0 | 0 | 0 | 0 | 0 | 2 (0.5%) | 1 (0.2%) | 4 (2%) | 1 (0.4%) |
| **Lung cancer** | 0 | 0 | 0 | 0 | 1 (0.2%) | 0 | 4 (1%) | 0 | 1 (0.4%) | 0 |
| **Breast cancer** | 0 | 0 | 4 (1%) | 3 (1%) | 3 (0.6%) | 3 (1%) | 4 (1%) | 5 (1%) | 3 (1%) | 3 (1%) |
| **GI cancer** | 0 | 1 (0.3%) | 1 (0.2%) | 1 (0.2%) | 0 | 0 | 0 | 0 | 1 (0.4%) | 0 |
| **Urogenital cancer** | 0 | 0 | 0 | 0 | 0 | 1 (0.2%) | 2 (0.5%) | 0 | 3 (1%) | 1 (0.4%) |
