# Supplementary material for: Disease prevalence and number of health care visits among members of a nationwide sports organization compared to matched controls
Source: BMC Public Health. 2021 Mar 6;21:455. doi: 10.1186/s12889-021-10466-9 (PMC7937278; doi:10.1186/s12889-021-10466-9)
Supplement: Supplementary file 5 — Additional file 5. Comparison between subjects with no or with one to five total number of health care visits over the two-year study period. [file 12889_2021_10466_MOESM5_ESM.docx]

| **Additional file 5. Comparison between subjects with no or with one to five total number of health care visits over the two-year study period.** | | | | | | |
| --- | --- | --- | --- | --- | --- | --- |
|  | **Controls (n=1713)** | |  | **Members (n=1769)** | |  |
|  | **No visit**  (n=644) | **1-5 visits** (n=1069) | P | **No visit** (n=327) | **1-5 visits** (n=1442) | P |
| **Males**, n (%) | 296 (46%) | 306 (29%) | <0.001 | 181 (55%) | 418 (29%) | <0.001 |
| **Age**, mean±SD | 49.5±17.0 | 52.5±14.5 | <0.001 | 51.3±13.7 | 52.4±14.8 | 0.16 |
| **Exercise frequency (data for members only)** | | | | | | |
| 1-2 times per week, n (%) | | | | 103 (32%) | 491 (34%) | 0.57 |
| 3-5 times per week, n (%) | | | | 203 (62%) | 849 (59%) |  |
| Every/almost every day, n (%) | | | | 21 (6%) | 102 (7%) |  |
| Number of visits represents total number of health care visits (primary and hospital visits). | | | | | | |
