# Supplementary material for: Disease prevalence and number of health care visits among members of a nationwide sports organization compared to matched controls
Source: BMC Public Health. 2021 Mar 6;21:455. doi: 10.1186/s12889-021-10466-9 (PMC7937278; doi:10.1186/s12889-021-10466-9)
Supplement: Supplementary file 6 — Additional file 6. Comparison between subjects with 1–20 or more than 20 total number of health care visits over the two-year study period. [file 12889_2021_10466_MOESM6_ESM.docx]

| **Additional file 6. Comparison between subjects with 1-20 or more than 20 total number of health care visits over the two-year study period.** | | | | | | |
| --- | --- | --- | --- | --- | --- | --- |
|  | **Controls (n=2370)** | |  | **Members (n=2687)** | |  |
|  | **1-20 visits**  (n=2001) | **>20 visits** (n=369) | P | **1-20 visits** (n=2449) | **>20 visits** (n=228) | P |
| **Males**, n (%) | 559 (28%) | 93 (25%) | 0.28 | 714 (29%) | 53 (23%) | 0.06 |
| **Age**, mean±SD (range) | 54.3±14.6 (20-86) | 55.7±15.9 (23-89) | 0.04 | 53.8±15.4 (20-92) | 52.3±17.9 (21-82) | 0.59 |
| **Exercise frequency (data for members only)** | | | | | | |
| 1-2 times per week, n (%) | | | | 850 (35%) | 79 (35%) | 0.89 |
| 3-5 times per week, n (%) | | | | 1435 (58%) | 131 (58%) |  |
| Every/almost every day, n (%) | | | | 174 (7%) | 18 (8%) |  |
| Number of visits represents total number of health care visits (primary and hospital visits). | | | | | | |
