# Supplementary material for: Disease prevalence and number of health care visits among members of a nationwide sports organization compared to matched controls
Source: BMC Public Health. 2021 Mar 6;21:455. doi: 10.1186/s12889-021-10466-9 (PMC7937278; doi:10.1186/s12889-021-10466-9)
Supplement: Supplementary file 7 — Additional file 7. Health care contacts per group and the type of health care provider profession. [file 12889_2021_10466_MOESM7_ESM.docx]

| **Additional file 7. Health care contacts per group and the type of health care provider profession.** | | |
| --- | --- | --- |
|  | **Controls (n=3014)** | **Members (n=3014)** |
| Total number of contacts (n) | 27658 | 22735 |
| Medical doctor, n (%) | 13196 (48%) | 10138 (45%) |
| Nurse, n (%) | 7438 (27%) | 6081 (27%) |
| Assistant nurse, n (%) | 1152 (4%) | 702 (3%) |
| Physiotherapist, n (%) | 2477 (9%) | 2364 (10%) |
| Chiropractor/naprapath, n (%) | 55 (0.2%) | 51 (0.2%) |
| Psychologist, n (%) | 711 (3%) | 648 (3%) |
| Other, n (%) | 2292 (8%) | 2458 (11%) |
| Percent denotes the proportion of the total number of contacts per group. One outlier control subject with >500 health care visits over two years and the matching member were removed from the total sample of 3015 subjects per group. | | |
